# The Impact of Living Arrangement Transitions on Depression Among Older Adults: How Transitions and Who Leaves Matter

**DOI:** 10.1093/geroni/igaf122.1448

**Published:** 2025-12-31

**Authors:** Yalu Zhang, Xintong Zhao, Meijun Wan

**Affiliations:** Stony Brook University, Stony Brook, New York, United States; Southwest Medical University, Luzhou, Sichuan, China (People’s Republic); Southwest Medical University, Luzhou, Sichuan, China (People’s Republic)

## Abstract

Many older adults experience transitions in living arrangements, particularly shifts from cohabitation to living alone due to widowhood or children moving out. These changes can impact mental health by increasing depression risks due to reduced social support. While prior research examines living arrangements and mental health, fewer studies focus on how different transitions to living alone affect depression, particularly between losing a spouse versus children. Using data from the China Health and Retirement Longitudinal Study (CHARLS) 2011–2020 and fixed-effects models, this study first examined the effect of transitioning from cohabitation to living alone on depression among older adults. Second, it compared the mental health effects between those who transition from living with a spouse to living alone and those who transition from living with children to living alone. Third, it further explored disparities in these effects between rural and urban older adults. The results indicate that transitioning to living alone is significantly associated with increased depressive symptoms. In particular, the older adults who transition from living with a spouse to living alone experience a notable rise in depression (β = 0.528, p < 0.001), while those transitioning from living with children to living alone exhibit an even greater increase in depression (β = 1.036, p < 0.001) in the national sample (N = 5,176). These patterns persist across rural and urban subsamples, with rural older adults (n = 4,126) experiencing more pronounced effects. These findings highlight the need for targeted social and mental health interventions that account for the nature of living arrangement transitions.

